# Supplementary material for: KPT330 improves Cas9 precision genome- and base-editing by selectively regulating mRNA nuclear export
Source: Commun Biol. 2022 Mar 17;5:237. doi: 10.1038/s42003-022-03188-0 (PMC8931069; doi:10.1038/s42003-022-03188-0)
Supplement: Supplementary file 2 — Supplementary Information (new) [file 42003_2022_3188_MOESM2_ESM.pdf]

Supplementary Information

KPT330 improves Cas9 precision genome- and base-editing by selectively regulating mRNA nuclear export

Yan-ru Cui[1,#], Shao-jie Wang[1,#], Tiancheng Ma[2], Peihong Yu[1,3], Jun Chen[4], Taijie Guo[2], Genyi Meng[2], Biao Jiang[1], Jiajia Dong[2,*], Jia Liu[1,5,6,7,*]

[1]Shanghai Institute for Advanced Immunochemical Studies and School of Life Science and Technology, ShanghaiTech University, Shanghai, 201210, China

[2]Key Laboratory of Organofluorine Chemistry, Center for Excellence in Molecular Synthesis, Shanghai Institute of Organic Chemistry, Chinese Academy of Sciences, 345 Ling-Ling Road, Shanghai 200032, China

[3]University of Chinese Academy of Sciences, Beijing, 100049, China

[4]College of Life Sciences, Zhejiang University, Hangzhou, Zhejiang, 310058, China.

[5]Shanghai Clinical Research and Trial Center, Shanghai, 201210, China

[6]Gene Editing Center, School of Life Science and Technology, ShanghaiTech University, Shanghai, 201210, China

[7]Guangzhou Laboratory, No. 9 XingDaoHuanBei Road, Guangzhou International Bio Island, Guangzhou 510005, Guangdong Province, China

[#]These authors contributed equally to this work

Correspondence should be addressed to J. L. (liujia@shanghaitech.edu.cn), J. D. (jiajia@sioc.ac.cn)

Supplementary Figures and Legends

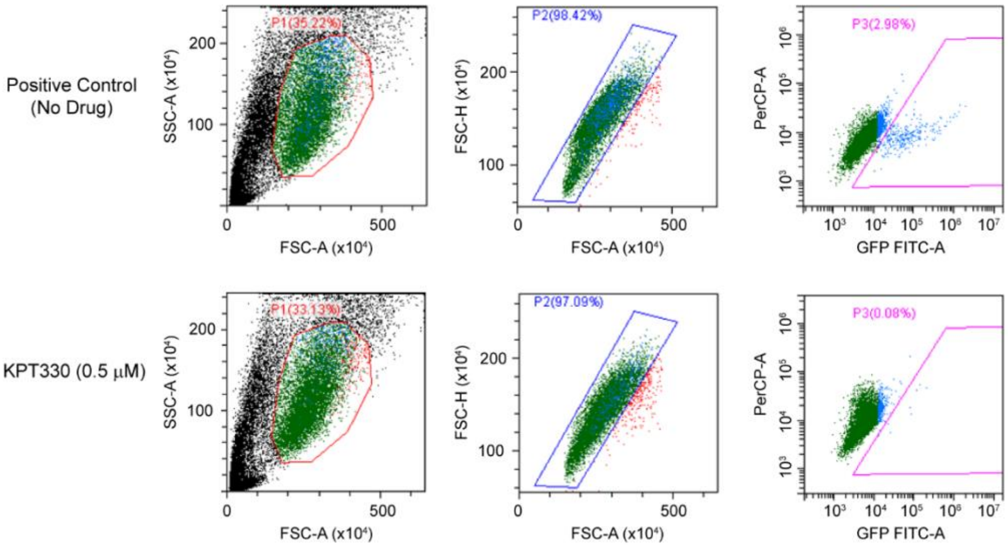

Supplementary Fig. 1: Flow cytometry analyses of the inhibition of CRISPR-activated EGFP fluorescence.

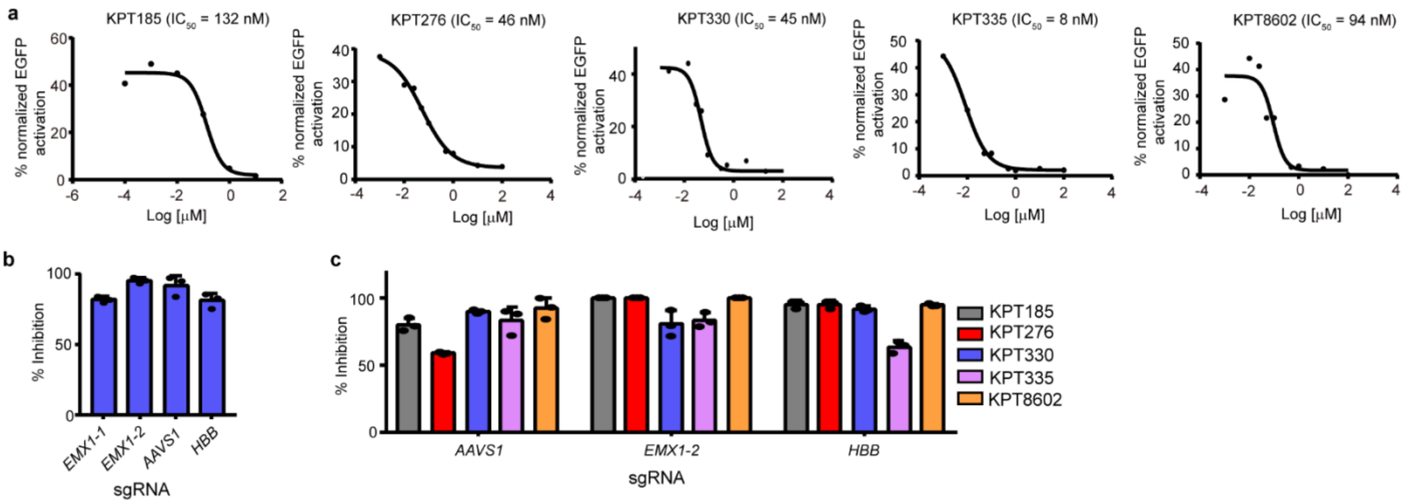

Supplementary Fig. 2: SINEs inhibit the activity of CRISPR-Cas9.

a Potency of SINEs on inhibiting CRISPR-mediated EGFP activation. The outlier data point in KPT335 is removed. **b** KPT330 at 10 μM efficiently inhibits the genome-editing activity of CRISPR-Cas9 at different endogenous sites in HEK293 cells, determined by T7E1 analysis. **c** SINEs at 10 μM inhibit CRISPR-Cas9 editing at endogenous sites in HEK293 cells, determined by T7E1 analysis. In (**b-c**), the data are shown as mean ± SD (n = 3).

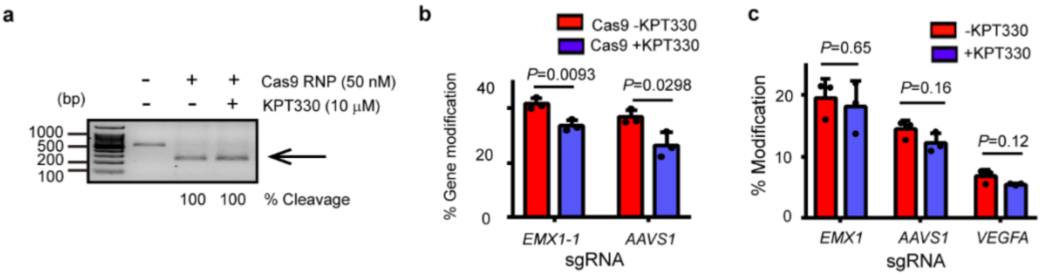

Supplementary Fig. 3: SINEs act as indirect inhibitors of CRISPR-Cas9

a Agarose gel (1.5%) showing that KPT330 does not inhibit the *in vitro* activity of Cas9-sgRNA RNP. The arrow indicates cleaved DNA product.

b The effects of KPT330 on Cas9-sgRNA RNP-mediated genome editing in Hela cells, determined by T7E1 analysis. KPT330 is supplemented into cell culture at 2 h following RNP nucleofection. **c** KPT330 at 0.5 µM does not affect the gene-editing activity of directly transfected Cas9 mRNA and sgRNA. In (**b-c**), the data are shown as mean ± SD (n = 3) and the significant difference between the drug-free and SINE treatment groups determined using Student's t test.

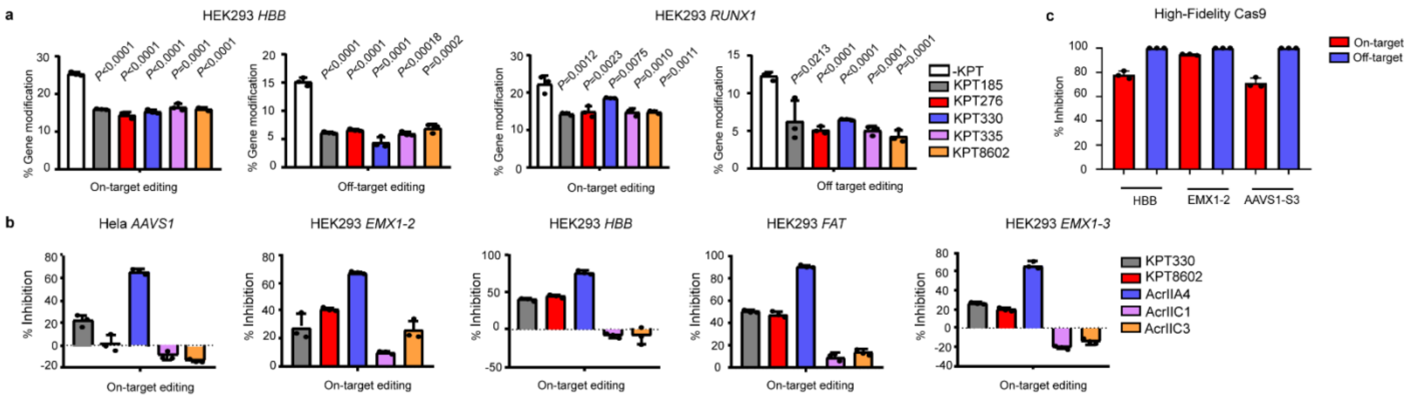

Supplementary Fig. 4: SINEs inhibit the gene-editing activity of CRISPR-Cas9 at different genomic sites.

a (Related to Figure 3a) The on- and off-target editing activity at *HBB* and *RUNX1* genes in the absence and presence of SINEs. **b** (Related to Figure 3b-f) Inhibition of on-target editing activity of CRISPR-Cas9 by SINEs and Acrs. **c** The inhibitory effects of high-fidelity Cas9 mutations on the on-target and off-target editing at the endogenous sites in HEK293 cells. % inhibition is calculated as the mutation rate of HF-Cas9 over that of wild-type Cas9. In (**a-c**), the data are shown as mean ± SD (n = 2 or 3) and the significant difference between the drug-free and SINE treatment groups determined using Student's t test.

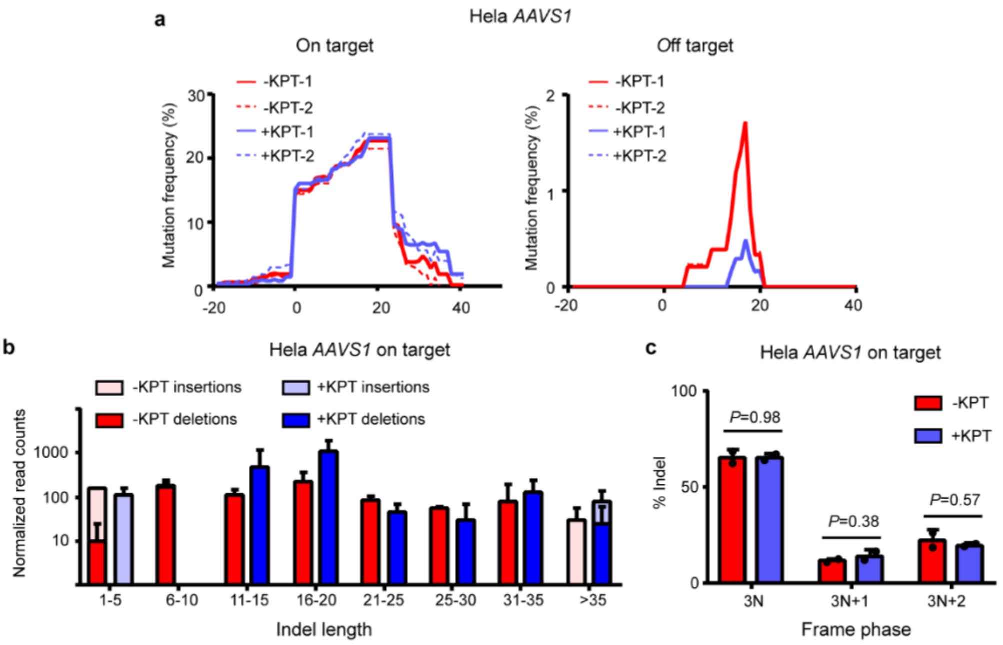

Supplementary Fig. 5 (Related to Fig. 3b): The effects of 0.5 μM KPT330 on the profile of Cas9-induced mutations

a Density plot showing the distribution of indel rates along CRISPR-Cas9-edited *AAVS1* site in Hela cells. **b** Distribution of indel length. The mean value of two biological replicates are shown. **c** Distribution of indel frame phase calculated as the length of indel modulus. In (**a**-**c**), the data are shown as mean ± SD (n = 2) and the significant difference between the drug-free and SINE treatment groups is determined using Student's t test.

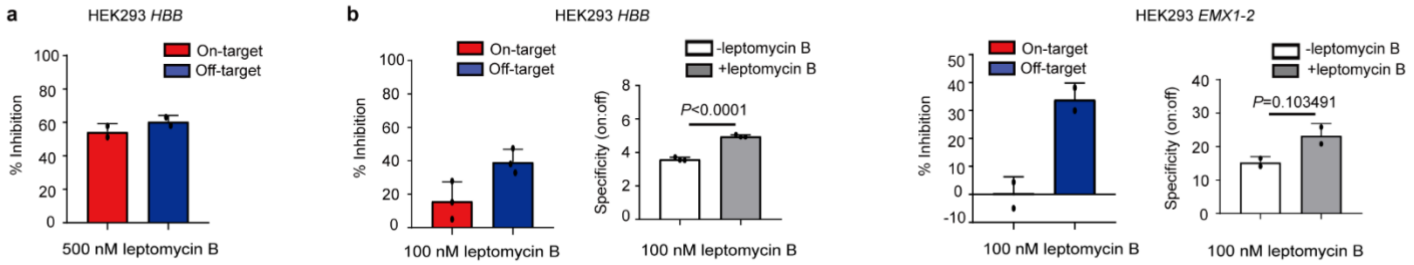

Supplementary Fig. 6: The effects of leptomycin B on the gene-editing activity and specificity of CRISPR-Cas9.

a The effects of 500 nM leptomycin B on the on- and off-target editing activities of CRISPR-Cas9 at the *HBB* site in HEK293 cells. **b** The effects of 500 nM leptomycin B on the specificity of CRISPR-Cas9 at the *HBB* and *EMX1* sites in HEK293 cells. In (**a-b**), the data are shown as mean ± SD (n = 2 or 3) and the significant difference between the drug-free and leptomycin B treatment groups determined using Student's t test.

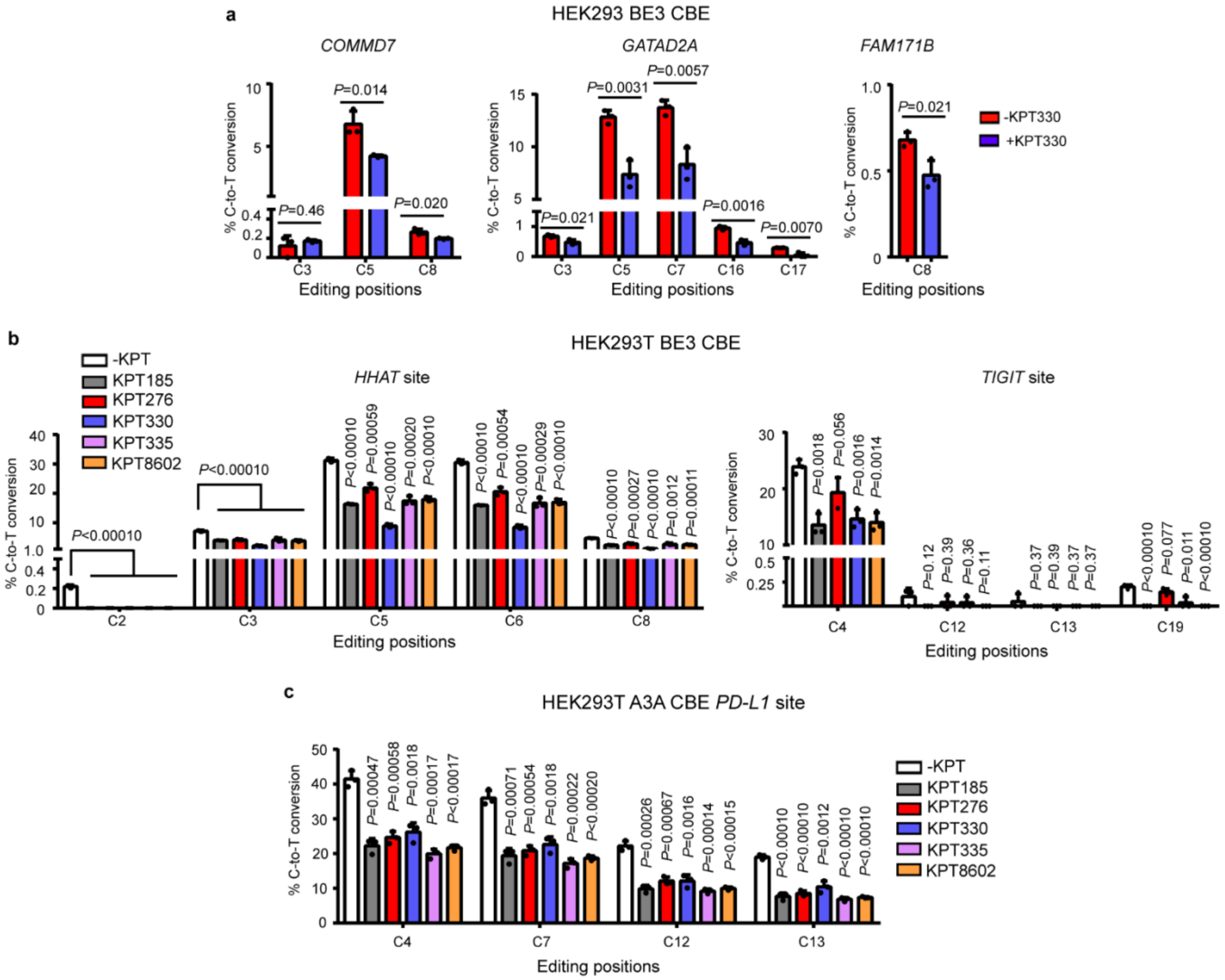

Supplementary Fig. 7: SINEs inhibit the activity of BE3 and A3A CBEs, determined by NGS analysis

a KPT330 at 0.5 μM inhibits BE3 CBE at various genomic loci. **b** SINEs at 0.5 μM inhibit BE3 CBE at *HHAT* and *TIGIT* sites in HEK293 cells. **c** SINEs at 0.5 μM inhibit A3A CBE at *PD-L1* site in HEK293 cells. In (**a**-**c**), the data are shown as mean ± SD (n = 3) and the significant difference between the drug-free and SINE treatment groups is determined using Student's t test.

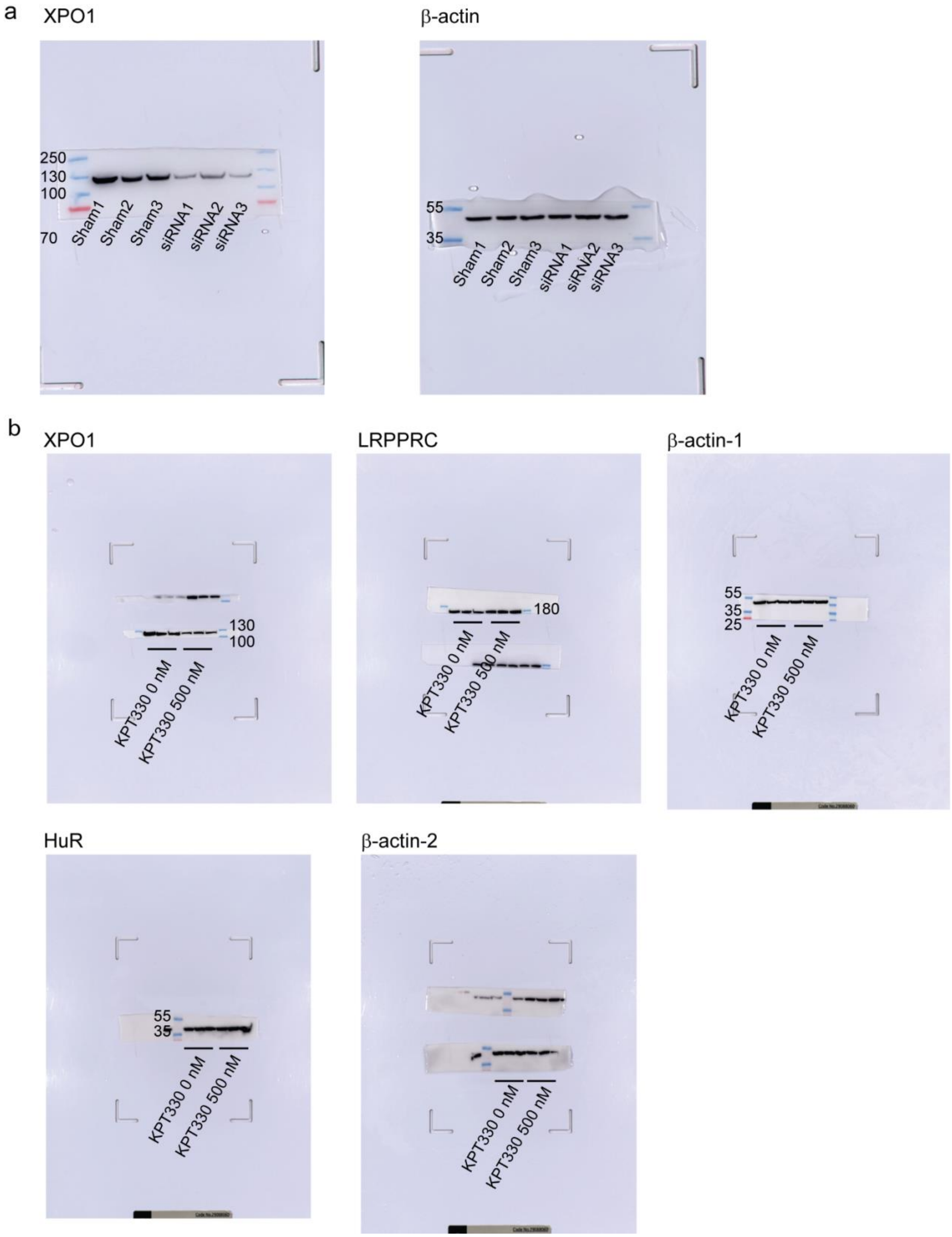

Supplementary Fig. 8: Uncropped images for WB.

a Related to Fig. 2c. **b** Related to Fig. 2g. XPO1, LRPPRC and β-actin-1 samples are from the same blot. HuR and the β-actin-2 are from another blot, where the blot is regenerated using striping buffer for β-actin staining.

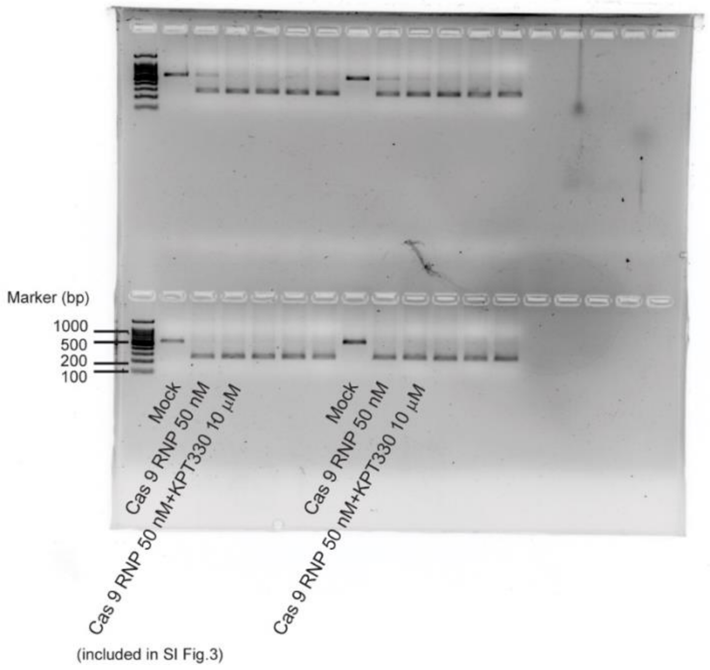

Supplementary Fig. 9: Uncropped agarose gel images (related to Supplementary Fig. 3a)

All the relevant samples and the representative images included in Supplementary Fig. 3a are indicated.

Supplementary Tables

Supplementary Table 1. Identification of Michael acceptor-bearing compounds for inhibition of EGFP activation by CRISPR-Cas9.

Internal Identifier	Structure	IC_{50} (μM)	CAS No. or Reference
32	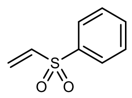	7.8	5535-48-8
49	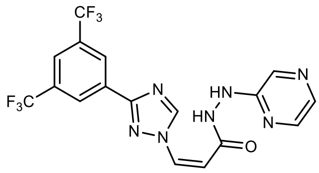	0.045	1393477-72-9 (KPT330)
75	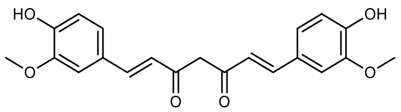	49	458-37-7
101	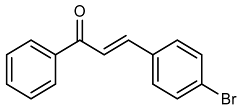	13	1774-66-9
104	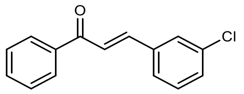	14	22966-13-8
110	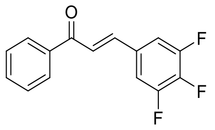	9.9	SI Ref. 1[1]
120	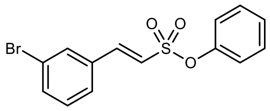	10	SI Ref. 2[2]

Supplementary Table 2. Targeted genomic sites.

Gene Target	Sequence (20 bp target with NGG PAM)
EMX1-1	GTCACCTCCAATGACTAGGGTGG
EMX1-2	GAGTCCGAGCAGAAGAAGAAGGG
EMX1-3[a]	GAGTCCGAGCAGAAGAAGAAGGG
FAT	GAGCTGCTTAAGCATTTCAAGGG
VEGFA-1	GGTGAGTGAGTGTGTGCGTGTGG
HBB	CTTGCCCCACAGGGCAGTAACGG
VEGFA-2	GGGTGGGGGGAGTTTGCTCCTGG
RUNX1	GCATTTTCAGGAGGAAGCGATGG
IL2RG	TGGTAATGATGGCTTCAACATGG
AAVS1	GGGAGGGAGAGCTTGGCAGGGGG
COMMD7	GGCACTGCGGCTGGAGGTGGGGG
GATAD2A	GGCACTCAGAACATTCCTGCTGG
TIGIT	GATCGAGTGGCCCCAGGTCCCGG
HHAT	GCCACCACCAGCAAAGAAGGCGG
FAM171B	ACAACAACAGCAAAAGCAGCTGG
PDL1	AGGCAGCAAATCCAGTTTGCCGG
FBN1^{T7498C}	CCAATGGTGTTAACACGTAGCGG

[a]Note: EMX1-3 has the same sgRNA sequence to EMX1-2, but is encoded by a different plasmid carrying GFP reporter for evaluation of the effects of plasmid backbone on genome editing.

Supplementary Table 3. siRNA sequences.

Gene Target	Sequence
XPO1-1-sense	5′-GGACAAGAGUCGACACAAUTT-3′
XPO1-1-antisense	5′-AUUGUGUCGACUCUUGUCCTT-3′
XPO1-2-sense	5′-GGCUGCUGAACUCUAUAGATT-3′
XPO1-2-antisense	5′-UCUAUAGAGUUCAGCAGCCTT-3′
XPO1-3-sense	5′-GGCUGUCAAUUCUCAUUGUTT-3′
XPO1-3-antisense	5′-ACAAUGAGAAUUGACAGCCTT-3′

Supplementary Table 4. pegRNA and nicking sgRNA sequences.

pegRNA	Spacer	3′ extension
RNF2	GTCATCTTAGTCATTACCTG	AACGAACATCTCAGGTAATGACTAAGATG
HEK3	GGCCCAGACTGAGCACGTGA	TCTGCCATCAAAGCGTGCTCAGTCTG
HEK4	GGCACTGCGGCTGGAGGTGG	TTAACCCCCCCCTCCAGCC
EMX1-4	ACTACGTGGTGGGCGCCGAG	AGCTGCTTCCGTTCGGCGCCCACCACG
COMP	GGCACGTTCCATGTGAACA	ATCCGTGACCGTGATCACATGGAACGT

Nicking sgRNA	Spacer
RNF2	GTCAACCATTAAGCAAAACAT
HEK3	GTCAACCAGTATCCCGGTGC
EMX1-4	GCCGGCCAGCTGCTTCCGTT
COMP	CGTAGAAGCTGGAGCTGTCC

Supplementary Table 5. Primers for PCR and QPCR.

Gene Target	Sequence
EMX1-T7E1-EXT-FWD	AGAGGAGCTAGGATGCAC
EMX1-T7E1-EXT-REV	TGAATTACCCTTGACCCC
EMX1-T7E1-INT-FWD	GGAGCAGCTGGTCAGAGGGG
EMX1-T7E1-INT-REV	AATCTACCACCCCAGGCTCT
AAVS1-T7E1-EXT-FWD	AGCCTGAGCGCCTCTCCT
AAVS1-T7E1-EXT-REV	TTGCTTTCTTTGCCTGGA
AAVS1-T7E1-INT-FWD	TGCTTCTCCTCTTGGGAAGT
AAVS1-T7E1-INT-REV	CGGTTAATGTGGCTCTGGTT
HBB-T7E1-FWD	TCACTTAGACCTCACCCTGTG
HBB-T7E1-REV	TATGGGACGCTTGATGTTTTCT
VEGFA-3-T7E1-FWD	CCAGATGAGGGCTCCAGATG
VEGFA-3-T7E1-REV	GTGAGGTTACGTGCGGACAG
EMX1-1-ON-NGS-FWD	CCTGAGTTTCTCATCTGTGC
EMX1-1-ON-NGS-REV	AGTCATTGGAGGTGACATCGATGT
EMX1-1-OFF-NGS-FWD	AAATGCCCAATCATTGATGC
EMX1-1-OFF-NGS-REV	GATTAACAGAGAGTCTGACACCTTTTAAGA
FAT-ON-NGS-FWD	AGGCTGGCATTAAGCAGGAA
FAT-ON-NGS-REV	TTTGGAGAGGATTTGGCTGGT
FAT-OFF-NGS-FWD	GGAAAATGAGCACTTAGCAATCAATTTG
FAT-OFF-NGS-REV	CTGTTTCTTTTTCTTGTTACTGCAGCA
AAVS1-ON-NGS-FWD	CTGGTGACACACCCCCATTT
AAVS1-ON-NGS-REV	CCAGGATCAGTGAAACGCACCAGACG
AAVS1-OFF-NGS-FWD	GCACCTTGCAAGAGAGGTAC
AAVS1-OFF-NGS-REV	TGACTAAGGCAGAGAGACCGAGGAAGC
COMMD7-NGS-FWD	AGATGGCTGACAAAGGCCG
COMMD7-NGS-REV	TTCAACCCGAACGGAGACAC
GATAD2A-NGS-FWD	CCTGGTGTCCTGCTCTCTTG

GATAD2A-NGS-REV	TCAGAAGGAAAGCGCAACCT
TIGIT-NGS-FWD	AGTGACCCAGGTCAACTGGG
TIGIT-NGS-REV	AGCTTTCTAGGACCTCCAGG
HHAT-NGS-FWD	ATGCAGCAGCAGGAGCATGACT
HHAT-NGS-REV	CCACTCCAGCTCCATTCTGATGCTAA
FAM171B-NGS-FWD	AGCCTCATCCAACAGCAGCAG
FAM171B-NGS-REV	AACGAGGATAGGAATGGATAGGGAAG
PDL1-NGS-FWD	ACTAGATACCTAAACTGAAAGCTTCCGC
PDL1-NGS-REV	CAGAGATACTGGGCCGTGGG
IL2RG-ON-NGS-FWD	TTGGGCGTCAGAATTGTCGT
IL2RG-ON-NGS-REV	ACAGAGGAAACGTGTGGGTG
RUNX1-ON-NGS-FWD	TGTCTTGGTTTTCGCTCCGA
RUNX1-ON-NGS-REV	TCTGCACCGAGGTGAAACAA
HBB-ON-NGS-FWD	GTCTCCACATGCCCAGTTTC
HBB-ON-NGS-REV	GCCCTGACTTTTATGCCCAG
HBB-OFF-NGS-FWD	TACCCTTTCCCGTTCTCCAC
HBB-OFF-NGS-REV	GCACAGCCAGATTTGGGAAT
FBN1-NGS-FWD	ACTCACCAATGCAGGACGTA
FBN1-NGS-REV	AGCTGCTTCATAGGGTCAGC
RNF2-NGS-FWD	ACGTCTCATATGCCCCTTG
RNF2-NGS-REV	ACGTAGGAATTTTGGTGGGACA
HEK3-NGS-FWD	ATGTGGGCTGCCTAGAAAGG
HEK3-NGS-REV	CCCAGCCAAACTTGTCAACC
HEK4-NGS-FWD	GAACCCAGGTAGCCAGAGAC
HEK4-NGS-REV	TCCTTTCAACCCGAACGGAG
EMX1-2-ON-NGS-FWD	TTTCTCATCTGTGCCCCTCC
EMX1-2-ON-NGS-REV	CCCTCGTGGGTTTGTGGTT
EMX1-2-OFF-NGS-FWD	TAGCTTTAAATGCCCAATCATTGATGC
EMX1-2-OFF-NGS-REV	GATTAACAGAGAGTCTGACACCTTTTAAGA

EMX1-3-NGS-FWD	AGTTTCTCATCTGTGCCCCT
EMX1-3-NGS-REV	ATCGATGTCCTCCCCATTGG
EMX1-3-OFF-NGS-FWD	TGGCATGGCAAGACAGATTG
EMX1-3-OFF-NGS-REV	CGCTTGTCCATGTCTAGGAA
EMX1-4-NGS-FWD	TCTGTACCTGCGTGTGTTGC
EMX1-4-NGS-REV	GGGGGTGATTACCTGCGTCT
COMP-NGS-FWD	AAAGGCCACTGCTCTCTTTCC
COMP-NGS-REV	TCTGCTTCCACATGACCACG
Actin-QPCR-FWD	CATGTACGTTGCTATCCAGGC
Actin-QPCR-REV	CTCCTTAATGTCACGCACGAT
Cas9-QPCR-FWD	CTCATTGTCTTCAGGGCTACC
Cas9-QPCR-REV	GAGAATGGTAGGAAGCGGATG
XPO1-QPCR-FWD	GCATTTCGTTCAGGTTCAGGG
XPO1-QPCR-REV	TGCACCAATCATGTACCCC

Supplementary References

1 Rendy, R., Zhang, Y., McElrea, A., Gomez, A. & Klumpp, D. A. Superacid-catalyzed reactions of cinnamic acids and the role of superelectrophiles. *J. Org. Chem.* **69**, 2340-2347 (2004).

2 Campbell, A. D. & Birch, A. M. Expedient syntheses of sulfonylhydantoins and two six-membered analogues. *Synlett*, 834-838 (2005).
