# Supplementary material for: KPT330 improves Cas9 precision genome- and base-editing by selectively regulating mRNA nuclear export
Source: Commun Biol. 2022 Mar 17;5:237. doi: 10.1038/s42003-022-03188-0 (PMC8931069; doi:10.1038/s42003-022-03188-0)
Supplement: Supplementary file 3 — Description of Additional Supplementary Files [file 42003_2022_3188_MOESM3_ESM.pdf]

Description of Additional Supplementary Files

File name: Supplementary Data 1
Description: Raw data for the results presented in this study.
