# Supplementary material for: KPT330 improves Cas9 precision genome- and base-editing by selectively regulating mRNA nuclear export
Source: Commun Biol. 2022 Mar 17;5:237. doi: 10.1038/s42003-022-03188-0 (PMC8931069; doi:10.1038/s42003-022-03188-0)
Supplement: Supplementary file 5 — Reporting Summary [file 42003_2022_3188_MOESM5_ESM.pdf]

nature research

Corresponding author(s): Jia Liu

Last updated by author(s): Jan 28, 2022

Reporting Summary

Nature Research wishes to improve the reproducibility of the work that we publish. This form provides structure for consistency and transparency in reporting. For further information on Nature Research policies, see our Editorial Policies and the Editorial Policy Checklist.

Statistics

For all statistical analyses, confirm that the following items are present in the figure legend, table legend, main text, or Methods section.

n/a	Confirmed	
☐	☒	The exact sample size (*n*) for each experimental group/condition, given as a discrete number and unit of measurement
☐	☒	A statement on whether measurements were taken from distinct samples or whether the same sample was measured repeatedly
☐	☒	The statistical test(s) used AND whether they are one- or two-sided<br>*Only common tests should be described solely by name; describe more complex techniques in the Methods section.*
☒	☐	A description of all covariates tested
☐	☒	A description of any assumptions or corrections, such as tests of normality and adjustment for multiple comparisons
☐	☒	A full description of the statistical parameters including central tendency (e.g. means) or other basic estimates (e.g. regression coefficient) AND variation (e.g. standard deviation) or associated estimates of uncertainty (e.g. confidence intervals)
☐	☒	For null hypothesis testing, the test statistic (e.g. *F*, *t*, *r*) with confidence intervals, effect sizes, degrees of freedom and *P* value noted<br>*Give P values as exact values whenever suitable.*
☒	☐	For Bayesian analysis, information on the choice of priors and Markov chain Monte Carlo settings
☒	☐	For hierarchical and complex designs, identification of the appropriate level for tests and full reporting of outcomes
☒	☐	Estimates of effect sizes (e.g. Cohen's *d*, Pearson's *r*), indicating how they were calculated

Our web collection on statistics for biologists contains articles on many of the points above.

Software and code

Policy information about availability of computer code

Data collection	Illumina Hiseq X Ten platform for high-throughput mutation sequence decoding. Paired end reads with 150 bp length were selected and cleaned to remove adaptor sequences and low quality paired reads. The following criteria were used to remove the low quality reads: (i) containing more than 10% 'N' s; (ii) containing more than 50% bases having low quality values (Phred score less than 5); (iii) duplicated reads. The coverage values were calculated using SAMTtools.
Data analysis	For analysis of CRISPR-Cas9-meidated genome editing, amplicons with less than 6 M read counts were excluded from the analyses.the barcodes of each sequence are extracted, the value of each base must be greater than 30 (>Q30); Short reads were aligned to the reference sequence by Bowtie2, Aligned reads were sorted by SAMtools1 and indel calling was performed by mpileup3 with maximum read depth per sample equal to the total reads mapped. VarScan v2.44 was used for the quality control of indels in mpileup output with a minimum variant frequency of [illegible]0.001, and a P value threshold of 0.05., Bam-readcount was applied to report the numbers of all types of nucleotide,For analysis of base editing (BE) and prime editing (PE) results, amplicon sequences were aligned to a reference sequence using CRISPResso2. Frequency, mean, and standard deviations were calculated using GraphPad Prism 8.Statistical analyses were performed using two-tailed Student's t test unless otherwise noted. Further details and references are provided in the methods. NGS data have been deposited into NCBI SRA database with the accession number PRJNA565327.

For manuscripts utilizing custom algorithms or software that are central to the research but not yet described in published literature, software must be made available to editors and reviewers. We strongly encourage code deposition in a community repository (e.g. GitHub). See the Nature Research guidelines for submitting code & software for further information.

Data

Policy information about availability of data

All manuscripts must include a data availability statement. This statement should provide the following information, where applicable:

- Accession codes, unique identifiers, or web links for publicly available datasets
- A list of figures that have associated raw data
- A description of any restrictions on data availability

Next-generation sequencing data have been deposited into NCBI SRA database with the accession number PRJNA565327

Field-specific reporting

Please select the one below that is the best fit for your research. If you are not sure, read the appropriate sections before making your selection.

☒ Life sciences ☐ Behavioural & social sciences ☐ Ecological, evolutionary & environmental sciences

For a reference copy of the document with all sections, see nature.com/documents/nr-reporting-summary-flat.pdf

Life sciences study design

All studies must disclose on these points even when the disclosure is negative.

Sample size	Sample size has been indicated for each experiment at place wherever relevant.
Data exclusions	Data exclusion criteria has been indicated for each experiment at place wherever relevant.
Replication	Biological replication
Randomization	N/A
Blinding	N/A

Reporting for specific materials, systems and methods

We require information from authors about some types of materials, experimental systems and methods used in many studies. Here, indicate whether each material, system or method listed is relevant to your study. If you are not sure if a list item applies to your research, read the appropriate section before selecting a response.

Materials & experimental systems

n/a	Involved in the study	
☐	☒	Antibodies
☐	☒	Eukaryotic cell lines
☒	☐	Palaeontology and archaeology
☒	☐	Animals and other organisms
☒	☐	Human research participants
☒	☐	Clinical data
☒	☐	Dual use research of concern

Methods

n/a	Involved in the study	
☒	☐	ChIP-seq
☐	☒	Flow cytometry
☒	☐	MRI-based neuroimaging

Antibodies

Antibodies used	rabbit anti-XPO1 monoclonal antibody (mAb; Cell Signaling, 46249S,WB:1:1000); anti-HuR mAb (Abcam, ab200342); anti-LRPPRC mAb (Abcam, ab205022); mouse anti-β-actin mAb with HRP conjugate (Cell Signaling, 12262S); HRP-linked anti-rabbit IgG antibody (Cell Signaling, 7074P2); IgG (Sigma-Aldrich, M7023)
Validation	N/A

Eukaryotic cell lines

Policy information about cell lines

Cell line source(s)	ATCC

Authentication	Verified by vendor
Mycoplasma contamination	Verified by qualified service company
Commonly misidentified lines (See ICLAC register)	N/A

Flow Cytometry

Plots

Confirm that:

☒ The axis labels state the marker and fluorochrome used (e.g. CD4-FITC).

☒ The axis scales are clearly visible. Include numbers along axes only for bottom left plot of group (a 'group' is an analysis of identical markers).

☒ All plots are contour plots with outliers or pseudocolor plots.

☒ A numerical value for number of cells or percentage (with statistics) is provided.

Methodology

Sample preparation	Cells were resuspended at 2.5*10^4 cells/100µL in PBS supplemented with 2% fetal calf serum (FCS buffer) and filtered through a 70-µm strainer, Data for at least 10000 cells were analyzed.
Instrument	CytoFLEX
Software	CytExpert
Cell population abundance	10^4 cells as the starting population;
Gating strategy	P1 represented starting cell population, P3 represented aimed cell population, FSC gating for FITC.

☒ Tick this box to confirm that a figure exemplifying the gating strategy is provided in the Supplementary Information.
